# Exploring Ward Registered Nurses' Emotional Responses When Caring for Clinically Deteriorating Patients: An Interview Study Informed by Affective Events Theory

**DOI:** 10.1111/jocn.70482

**Published:** 2026-08-01

**Authors:** Karen Davey, Duncan Smith

**Affiliations:** ^1^ Imperial College Healthcare NHS Trust Charing Cross Hospital London England UK; ^2^ Department of Interprofessional Healthcare, School of Health and Medical Sciences City St George's, University of London London England UK; ^3^ University College London Hospitals NHS Foundation Trust London England UK

**Keywords:** burnout, clinical deterioration, coping skills emotional regulation, emotional response, nursing workforce, patient safety, Qualititve research

## Abstract

**Aim:**

To explore ward‐based registered nurses' emotions, emotion‐driven behaviours, and the perceived impact of these emotions on caring for clinically deteriorating patients.

**Background:**

Clinical deterioration in ward patients is frequently missed or poorly managed despite widespread implementation of Early Warning Score systems. Behavioural and systemic factors may contribute to these failures; however, emotional influences remain underexplored.

**Design:**

Descriptive interview study underpinned by Affective Events Theory.

**Methods:**

Registered nurses from a United Kingdom teaching hospital with experience caring for clinically deteriorating patients were purposively sampled. Semi‐structured interviews were conducted using a topic guide informed by the seven Affective Events Theory constructs. Interviews were audio‐recorded, transcribed verbatim, and analysed using a combined inductive thematic approach and deductive mapping to theoretical constructs.

**Results:**

Five themes were identified and mapped to six of the seven Affective Events Theory constructs: *work environment, work events, affective reactions, work attitudes, affect‐driven behaviours and judgement‐driven behaviours*. The dispositions construct did not emerge as a distinct theme. Participants predominantly described negative emotional responses. Short‐term coping strategies, including emotional distancing, supported care delivery but were perceived to increase longer‐term burnout risk. Affect‐driven behaviours included heightened vigilance and increased bedside presence; however, reluctance to leave deteriorating patients conflicted with established escalation protocols. Judgement‐driven behaviours shaped longer‐term attitudes, including reduced motivation and intentions to leave nursing.

**Conclusions:**

Emotional responses to clinical deterioration shaped immediate nursing behaviours that were both beneficial and potentially detrimental to patient care, while also influencing longer‐term professional attitudes.

**Patient Contribution:**

Two patient and public involvement advisors reviewed the interview topic guide before data collection and contributed to the development of questions exploring the therapeutic relationship between nurses and patients.

## Abstract (highlights)

**Summary:**

- What does this paper contribute to the wider global clinical community?
  ○ Offers insight into an under‐researched topic on how nurses' emotional behaviours shape management of deteriorating patients and adherence to Early Warning Score systems.
  ○ Highlights the need for strategies to address burnout and enhance resilience when managing deteriorating patients, supporting nurse retention and a sustainable global workforce.

**Key Highlights:**

- Clinically deteriorating patients evoke predominantly negative emotions, particularly anxiety, fear and frustration in ward nurses.
- Emotions shape escalation decisions and other patient safety behaviours during deterioration events.
- Emotional distancing provides short‐term coping but may increase the risk of longer‐term psychological harm.
- Frequent exposure to deterioration may contribute to burnout, altered professional identity and nurse retention challenges.

## 1 Introduction

Clinical deterioration refers to a decline in a patient's physiological state, increasing the risk of morbidity and mortality (Jones et al. 2013). Clinical deterioration remains a significant international patient safety challenge, with failures to recognise and respond appropriately continuing to pose a persistent threat to patients worldwide. For example, in the United Kingdom, timely recognition and management could prevent nearly 5000 in‐hospital deaths annually (NHS England 2023). While global incidence of unrecognised deterioration is difficult to quantify, due to international variation in definitions and reporting, in‐hospital cardiac arrest rates range from approximately 1–10 per 1000 admissions (Penketh and Nolan 2022). Many of these events are preceded by identifiable physiological deterioration, highlighting ongoing opportunities for earlier recognition and intervention.

Early Warning Scores (EWS) are used internationally to support early recognition and management of patient deterioration through standardised monitoring and escalation of care protocols (Lyons et al. 2018). Routine vital signs (e.g., heart rate, respiratory rate, blood pressure) are recorded and used to calculate an EWS indicating a patient's risk of deterioration. When this score reaches a predefined threshold, or when staff have clinical concerns despite a low score, care should be escalated to an appropriate responder. The responder may be a ward‐based clinician or a member of a specialist team, such as a Rapid Response Team (RRT)—also known internationally as a Medical Emergency Team, Critical Care Outreach Team or, in some organisations, a Medical Response Team—which provides urgent assessment and intervention for deteriorating patients (Zhang et al. 2024). Despite variations in RRT composition, systematic review findings indicate a reduction in unexpected cardiac arrests and mortality associated with these teams (Zhang et al. 2024). Yet, despite international implementation of EWS tools and RRTs, delays and failures in recognising and responding to patients with clinical deterioration persist (Considine et al. 2023). Research conducted across the world demonstrates the multifactorial nature of the problem which includes rigid hierarchies, poor safety culture, ineffective communication, junior staff disempowerment and variable knowledge and skills amongst ward staff (Burke et al. 2022; Chua et al. 2017). These findings suggest the barriers to effective recognition of clinical deterioration and escalation of care are intertwined with deeper behavioural processes (Smith et al. 2021).

Emotional experiences; that is, the interactions of cognitive appraisal, physiological arousal and action readiness (Frijda 2009), can influence nurses' behaviours and decision‐making when delivering clinical care (LeBlanc et al. 2015). Caring for clinically deteriorating patients can place an emotional burden on nurses (Dresser et al. 2023), which may inform their behaviour in clinical practice. Furthermore, prolonged or repeated psychological exposure to work stressors, such as caring for clinically deteriorating patients, has been linked with burnout and attrition (Archer et al. 2024). Burnout includes emotional exhaustion, depersonalisation and reduced efficacy which can compromise clinical performance and increase the likelihood of nurses leaving the profession (Rushton et al. 2015). With a projected global shortage of 4.1 million nurses by 2030 (World Health Organization 2025), factors that influence staff retention, such as the emotional demands of caring for clinically deteriorating patients, warrant exploration to better understand how workforce sustainability may be ensured.

In two empirical studies conducted in the UK (Smith et al. 2021) and Australasia (Walker et al. 2021), a 14‐domain theoretical framework of behaviour change (Cane et al. 2012) was used to explore barriers and enablers to nurses recognising and responding to clinical deterioration. Smith et al. (2021) reported that nurses' emotions both supported and hindered effective management, whereas Walker et al. (2021) highlighted that emotions, such as fear of criticism from the RRT, impede nurses from taking appropriate action, such as escalating care. Whilst existing studies offer preliminary insights into nurses' emotional experiences when caring for clinically deteriorating patients, their broad theoretical scope may have limited depth of exploration. Consequently, a more focused and in‐depth understanding of the emotional factors shaping escalation practices is required to advance knowledge in this area and inform the development of tailored improvement strategies for clinical deterioration management and staff wellbeing.

### 1.1 The Study

The aim of this study was to explore emotions experienced by registered nurses when caring for a clinically deteriorating patient and the perceived impact of these emotions on their behaviour. Specific objectives were to:

- Report the range of emotions experienced by nurses when caring for a clinically deteriorating patient.
- Use theory to deepen understanding of how emotions drive nurses' behaviours when caring for clinically deteriorating patients, and how these emotions influence attitudes towards their future careers.

## 2 Methods

### 2.1 Design

A descriptive qualitative study using semi‐structured interviews informed by Affective Events Theory (AET) (Weiss and Cropanzano 1996). Reporting was guided by the Consolidated Criteria for Reporting Qualitative Research (COREQ) checklist (Tong et al. 2007).

### 2.2 Theoretical Framework

Affective Events Theory (AET) was selected as it provides a psychological framework for understanding how nurses' emotional experiences shape subsequent behavioural responses. AET has been widely applied across organisational settings, including healthcare, to examine how workplace events trigger emotional reactions that influence attitudes and performance (Christensen et al. 2023). AET provides a useful lens for conceptualising how nurses' emotions influence their behaviour in high‐pressure situations (Christensen et al. 2023) such as providing care to a clinically deteriorating patient.

AET comprises seven interrelated constructs (Figure 1). The theoretical assumptions underpinning the framework are *work events*, such as caring for a clinically deteriorating patient, trigger *affective reactions* that is, an emotional response, which, in turn, influences *affect‐driven behaviour* (e.g., having heightened focus on a specific task, coping behaviours or a reactive outburst during an emotionally charged situation). Over time, these emotional experiences can shape *work attitudes*, such as job satisfaction and motivation, which may ultimately *affect judgement‐driven behaviours*, including absenteeism or the decision to leave a post or a profession. AET also accounts for the moderating effects of contextual factors, such as the *work environment* and an *individual's disposition*, for example traits such as whether the person is introverted or extroverted (Weiss and Cropanzano 1996).

**FIGURE 1:**
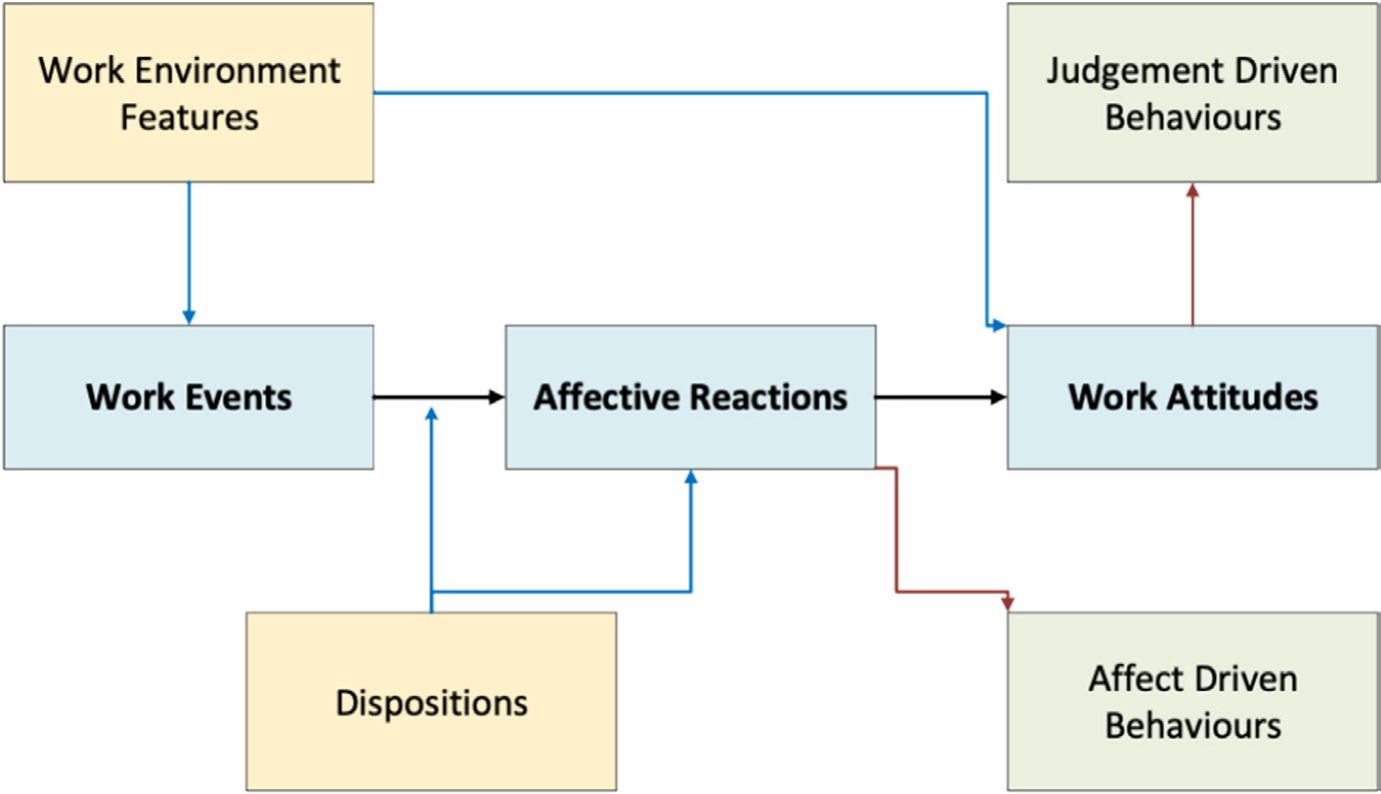
Affective Events Theory: Macro Structure (Weiss and Cropanzano 1996, 12). [Colour figure can be viewed at wileyonlinelibrary.com]

### 2.3 Study Setting and Recruitment

The study was conducted across three metropolitan teaching hospitals in the UK, all within the same healthcare organisation, covering services such as acute medicine, general surgery, neurosurgery, major trauma and cardiology. All three hospitals use the same EWS tool and have a Rapid Response Team (RRT) comprised of medical and critical care outreach (CCO) practitioners to support care of clinically deteriorating patients.

Registered nurses of varying bands were purposively recruited if they had cared for a deteriorating patient in the previous 28 days, a period chosen to optimise recall (Spearing and Wade 2022). Eligible participants worked in inpatient wards with adult patients (≥ 18 years). Nurses in emergency or critical care units were excluded as the processes and procedures for caring for clinically deteriorating patients in these areas differ from those of the wards.

Nurses who had worked with the local CCO team during a deterioration event, triggered by EWS criteria, clinical concern, or RRT activation, received a participant information sheet (PIS) from the responding CCO member at an appropriate time point after the clinically deteriorating patient had been made safe. The PIS provided contact information for the primary researcher (KD), enabling interested participants to opt in to the study. Interested participants were then offered a slot for a semi‐structured interview at a mutually convenient time.

A rich, in‐depth understanding of an underrepresented phenomenon was prioritised over diversity of perspectives. Accordingly, a sample size of six was considered sufficient and methodologically appropriate, consistent with the concept of information power (Malterud et al. 2016). Given the focused research aim, the use of a clearly defined theoretical framework, and the limited pool of eligible participants, this sample size was deemed adequate to address the study aim. The primary researcher's (KD) relatively limited experience in conducting semi‐structured interviews, having previously undertaken only one interview‐based study, was also considered when assessing the study's information power. To strengthen the credibility and trustworthiness of the findings, several measures were implemented, including a pilot interview, review of the interview topic guide by a more experienced qualitative researcher (DS), regular debriefing sessions, and ongoing reflexive practice. Although DS did not participate in data collection, their experience as a clinician working in critical care outreach and as a researcher enabled them to provide methodological oversight and contribute to critical discussion throughout the analytical process.

### 2.4 Participants

No participants withdrew at any stage of data collection or analysis. Due to multiple members of the CCO team distributing PIS to eligible nurses, the total number of nurses approached for recruitment could not be determined.

Participants ranged in age from 26 to 60 years and had between 4 and 15 years of post‐registration nursing experience. The sample comprised four female and two male registered nurses.

### 2.5 Data Collection

Semi‐structured interviews were chosen to facilitate in‐depth exploration of nurses' emotional experiences when caring for clinically deteriorating patients. This approach provided the flexibility to probe and clarify responses while ensuring key topics were explored consistently across interviews. An interview topic guide, informed by the constructs of Affective Events Theory (AET), provided a flexible framework for discussion and the exploration of issues raised by participants. The guide was piloted with a registered nurse from an eligible ward. Following piloting, the interview questions were revised to encourage a deeper exploration of participants' lived experiences rather than focusing on broad attitudes. Two patient safety partners from the organisation's deteriorating patient steering group were involved as patient and public involvement advisors. Their involvement was based on their role within the steering group and their knowledge and experience relating to the care of deteriorating patients. The advisors reviewed the interview guide before data collection and provided feedback on the relevance and comprehensiveness of the questions. Their recommendations resulted in the inclusion of questions exploring the therapeutic relationship between nurses and patients.

Semi‐structured interviews were conducted in a private room at the participant's workplace, with only the researcher (KD) and the participant present. Interviews were audio‐recorded, transcribed verbatim and anonymised. Field notes were documented during and following the interviews. No repeat interviews were conducted, and participants did not review transcripts or emergent themes.

### 2.6 Data Analysis

Data analysis followed a combined inductive and deductive approach. First, data were inductively analysed, informed by the procedure for thematic analysis reported by Braun and Clarke (2006). The exact procedure for inductive analysis was as follows: (1) transcripts were read and re‐read and initial thoughts, ideas, meanings, and patterns written down (by KD) as analytic memos; (2) using initial notes codes were manually generated from across the corpus of data. Data relevant to each code were collated; (3) codes were manually interrogated to identify emerging patterns and themes, and a ‘thematic map’ was developed to show potential relationships between the data; (4) sub themes were generated by KD and themes and subthemes were manually reviewed and checked (by KD and DS) for coherence to ensure they adequately represented the subordinate codes and illustrative participant quotes; (5) themes were then deductively mapped to the seven constructs of the AET. The primary researcher (KD) performed the mapping, which was reviewed by a second researcher (DS) and refined through consensus discussion.

KD maintained a reflexive journal throughout the interview process and data analysis. This personal record was used to document thoughts, feelings, observations, and reflections on how personal and professional experiences, assumptions, and characteristics may have influenced the research process. The journal also supported the ongoing development of interviewing skills and reflexive awareness (Yip 2024).

### 2.7 Ethical considerations

Favourable ethical approval to conduct the research was granted by City St Geroge's University Research Nursing Committee (ID: ETH2324‐24690), the Health Research Authority (ID: 21/PR/0305), and Imperial College Healthcare Trust Hospitals' Research Governance and Integrity Teams (local reference: 176789). All participants provided prospective informed written consent, including consent for the use of anonymised quotations.

The primary researcher (KD) occupied an insider position as a critical care outreach nurse within the same organisation as the participants. KD had previously worked clinically alongside, or provided formal education to, five of the six participants. While this familiarity facilitated rapport and may have enabled participants to share rich and detailed accounts of their experiences (Yip 2024), it also introduced ethical challenges.

The researcher‐participant relationship required careful consideration because the critical care outreach role represented a more senior professional position than those held by the participants. This raised the possibility of perceived power imbalances, social desirability bias, or concerns that participation and responses might influence professional relationships (Bergen and Labonté 2020). To mitigate these risks, participation was entirely voluntary, participants were informed that the study aimed to understand their experiences rather than evaluate their clinical practice, and assurances were provided regarding confidentiality, anonymity, and the absence of any impact on employment or professional standing. The researcher was not involved in any of the clinical events discussed and held no managerial responsibility for any participant.

### 2.8 Rigour and Reflexivity

The primary researcher's professional background and prior relationships with participants had the potential to influence data collection and interpretation through pre‐existing assumptions and shared understandings. To address this, reflexivity was actively maintained throughout the study through regular self‐reflection and the use of the reflexive journal.

Analytical rigour was enhanced through systematic coding procedures, transparent documentation of analytical decisions, and regular discussion of coding and theme development with a second researcher (Morse 2015). As DS was not involved in data collection, was not employed within the study site, and had no prior relationship with the participants or knowledge of the clinical events discussed, they were able to provide an independent perspective that challenged KD's assumptions and supported reflexive engagement with the data.

## 3 Findings

Semi‐structured interviews were conducted with six registered nurses between February and March 2025. Each interview lasted approximately 45 min, with an additional 5 min allocated prior to commencement for rapport building. Following the sixth interview, KD and DS agreed that no new substantive insights were emerging, which further supported the adequacy of the sample size.

Four participants reported caring for multiple deteriorating patients within the preceding 28 days. Two of the clinical events reflected upon involved missed escalation of care, in which the EWS met criteria for RRT activation but escalation did not occur.

From the inductive analysis, five core themes and 17 sub‐themes were generated. The core themes were:

1. Different trigger events across the continuum.
2. Coping strategies and resilience.
3. Emotional distancing.
4. Impact of emotions on behaviour.
5. Impact on identity, work, and career progression.

These core themes represent the primary analytic structure of the findings, with sub‐themes providing further detail and nuance within each theme. The themes and sub‐themes are presented in Figure 2. In the subsequent narrative, findings are presented under the headings of these core themes to reflect their central role in the analysis.

**FIGURE 2:**
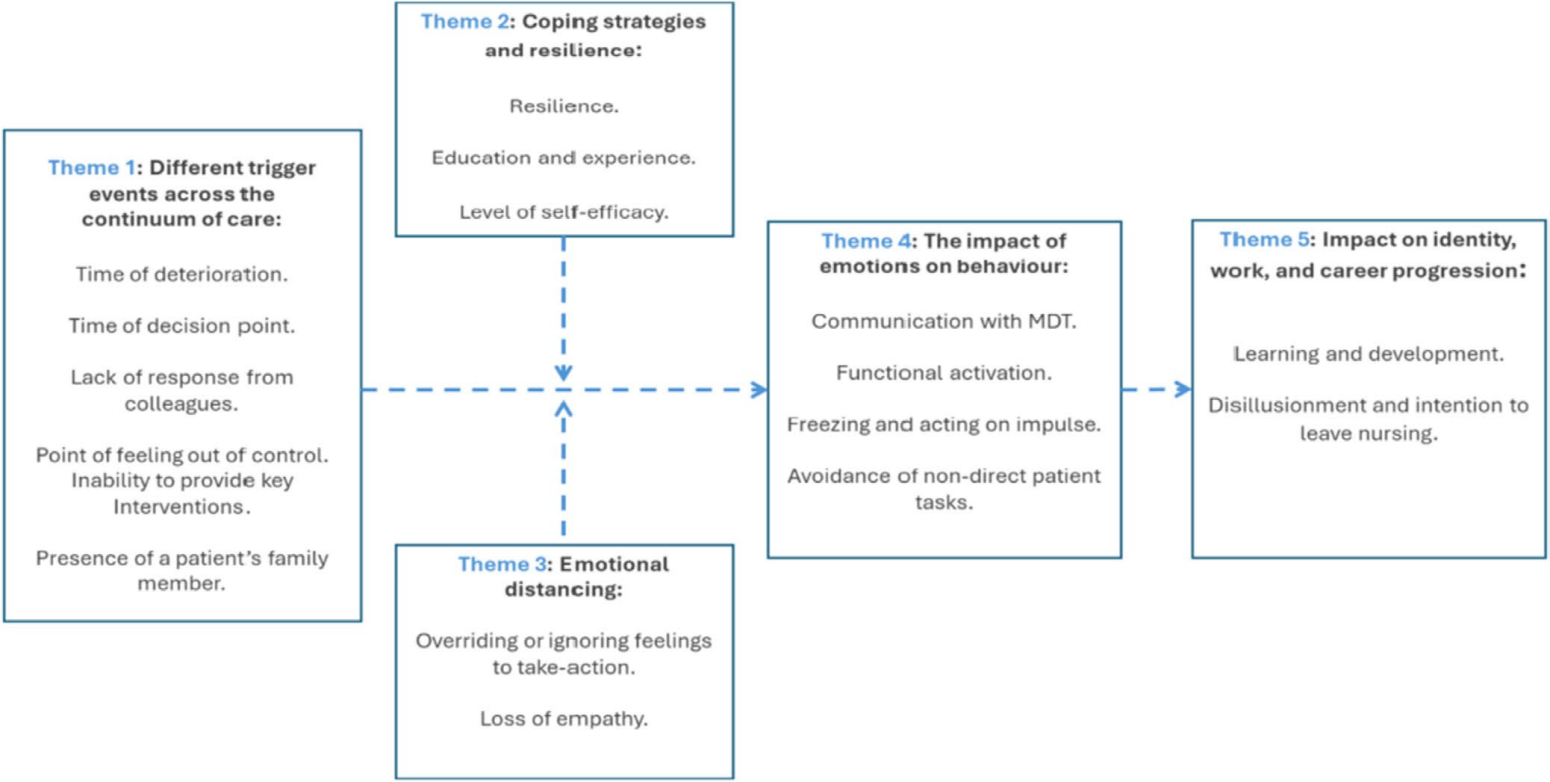
Overview of themes, subthemes, and their relational connections. [Colour figure can be viewed at wileyonlinelibrary.com]

The five core themes are mapped onto six of the seven AET constructs as displayed in Figure 3. The dispositions construct did not align with any theme or sub‐theme. In AET, individual dispositions refer to stable personal traits (e.g., optimism or extraversion) that may shape emotional and behavioural responses to work‐related events. During analysis, data were examined for evidence of this construct; however, it did not emerge as a distinct theme.

Participants' accounts instead focused on situational and relational factors, particularly trigger events, coping strategies, and the impact of emotions on behaviour and professional identity. While some accounts may reflect individual differences in response style, these were not sufficiently developed or consistent to form a separate analytic category. The dispositions construct is therefore not represented as an independent theme but is considered in relation to coping processes in theme 2.

**FIGURE 3:**
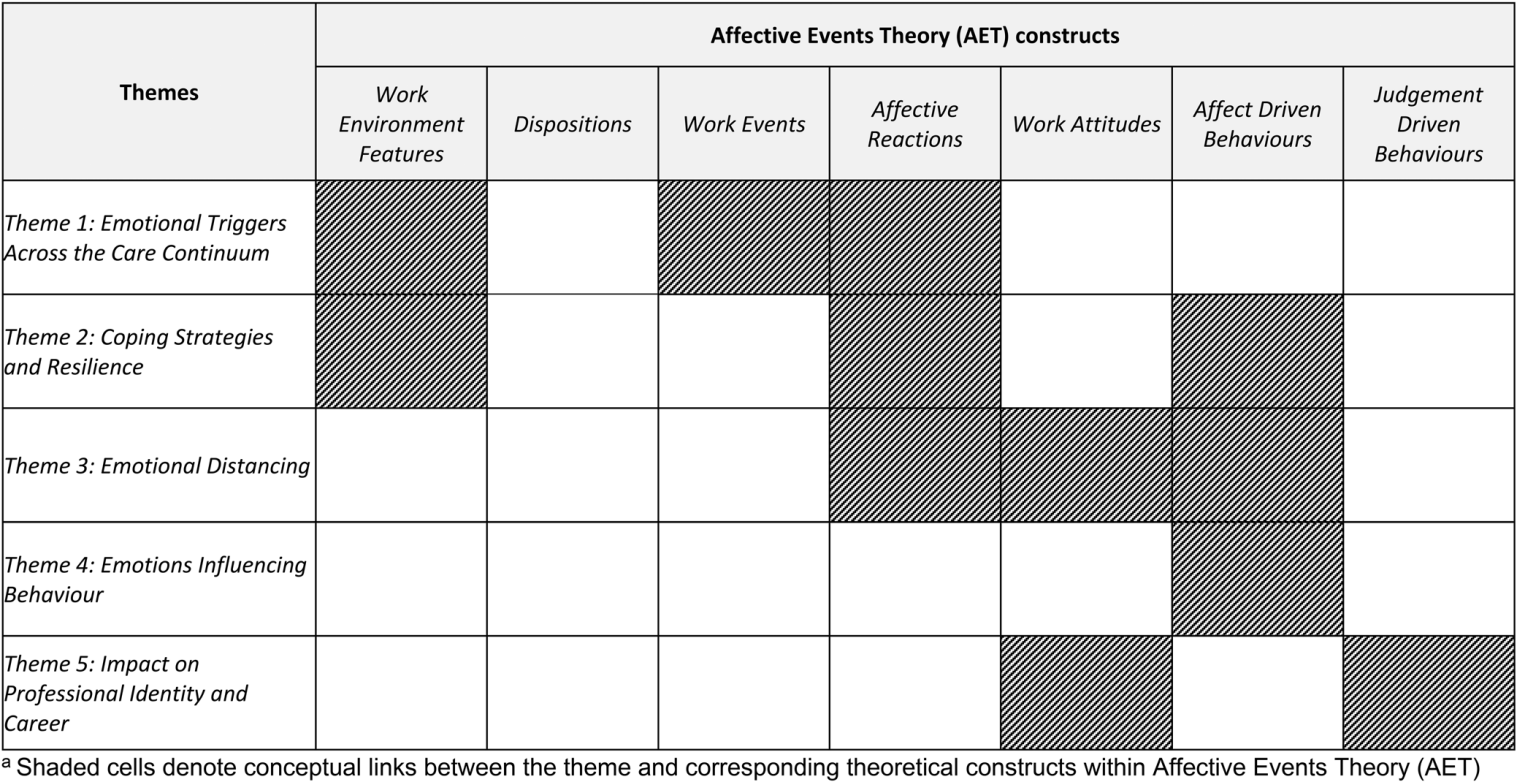
Links between inductively generated themes and theoretical constructs from the Affective Events Theory (AET).

### 3.1 Theme 1—Different Trigger Events Across the Continuum

Participants described patient deterioration as a workplace event that elicited emotional responses throughout different stages of care. Accounts revealed a range of emotions, reflecting the individual and subjective nature of affective workplace experiences proposed by AET. While participants' responses varied, anxiety and frustration emerged as the most commonly reported emotions. These emotional reactions were frequently associated with the recognition of patient deterioration and the requirement to make timely clinical decisions:

“It makes it a bit more anxious because you're taking the decision on yourself [to start oxygen therapy on a hypoxemic patient].” – RN5.

“We thought that we were in control of the situation … A few minutes. Nope. … you got stressed because you lost control … you feel helpless” – RN1.

Participants also described emotional reactions triggered by factors beyond their control that impeded effective management of the patient. These included insufficient support from other nursing staff or medical colleagues, or lack of equipment. One participant reported feeling a sense of responsibility regarding the lack of a multi‐disciplinary team (MDT) management plan during handover, despite lacking the authority to address the issue when doctors were unresponsive:

“… you're quite isolated. Because it is a night shift, and no one is listening to you … at least give me a [vital sign] parameter because during handover, they kill you without it [referring to documentation of a clinical parameter]” – RN4.

### 3.2 Theme 2: Coping Strategies and Resilience

In line with AET, the emotional impact of clinically deteriorating patient events was not experienced uniformly amongst participants; rather, their prior experience, education, and resilience appeared to influence how these events were interpreted and managed.

Specifically, their clinical exposure and participation in education focused on caring for clinically deteriorating patients shaped both the intensity of these emotional reactions and their perceived ability to deliver effective care.

“I was not scared… we see this all the time.” – RN3.

“This is the good thing about education… before I was scared to go see the patient” – RN6.

Several participants reported confidence in engaging with the MDT due to a strong sense of self‐efficacy. The same participants also reported that their resilience had strengthened over time, particularly because of repeated exposure to acutely unwell and clinically deteriorating patients during the COVID‐19 pandemic, which enhanced their ability to remain composed and effective during deterioration events:

“I've become resilient, especially after COVID Oh, I cried then. I even ran out once during my shift.” – RN3.

Participants' experiences and education influenced their affective reactions in a manner similar to the dispositional influences described in AET. However, as AET conceptualises dispositions as relatively stable traits, the findings demonstrate only a partial alignment with the theory, given that participants attributed their responses to resilience, education, and prior experience; factors developed through experience rather than fixed personal characteristics.

### 3.3 Theme 3: Emotional Distancing

Most participants acknowledged that emotions experienced during emergencies could be counterproductive. However, two participants described routinely using emotional‐distancing strategies to function effectively and protect themselves from emotional distress:

“You have to deal with it in a calm manner… if you are shaky, you cannot make a decision.” – RN6.

Emotional detachment was viewed as a necessary coping mechanism during traumatic events. One participant described automatically setting emotions aside in the moment:

‘It's been traumatic for me, but now I do everything without feeling … sometimes you have to be invasive. So, I do that, I don't care … it's how you protect yourself’, I feel lucky if I am honest, because are you able to jump on a baby and do the CPR, without thinking?’ – RN4.

However, the same two participants also reflected on the longer‐term consequences of repeated emotional distancing, reporting a diminishing sense of genuine empathy and, in some cases, feelings of dehumanisation:

“I need to empathise with them [patients] … after too many events it feels like automatic. It's just an empathy, just out of show … but you don't feel inside, it's numb inside … I don't feel human.” – RN6.

Consistent with AET, participants' accounts suggested that clinically deteriorating patient events triggered emotional responses that subsequently influenced coping behaviours. Emotional distancing emerged as a prominent strategy, enabling participants to maintain professional functioning while managing the emotional demands associated with patient deterioration. Although the participants felt this response helped facilitate care delivery in the immediate situation, they also reflected on the potential costs of repeatedly employing emotional distancing, including reduced empathy, emotional exhaustion and changes in self‐perception. In line with AET, these emotional experiences and coping responses appeared to have implications beyond the immediate event, shaping participants' longer‐term attitudes towards themselves and their careers. These longer‐term effects are explored further in Theme 5.

### 3.4 Theme 4: Impact of Emotions on Behaviour

Participants frequently described anxiety and fear as influencing how they responded to clinically deteriorating patient events. Rather than being solely detrimental, these emotions were often perceived as facilitating adaptive behaviours, including increased vigilance, assertive communication and rapid decision‐making. This is consistent with AET, which proposes that affective reactions to workplace events can shape subsequent behavioural responses that may be either functional or dysfunctional for work performance.

“I'm more assertive when I'm anxious… I make decisions quicker.” – RN5.

“You are more alert… it makes you organised” – RN1.

One participant reflected on the dual nature of their emotional response, noting that while fear facilitated automatic actions such as calling for help, it simultaneously impeded their ability to initiate CPR. In this instance, fear triggered a momentary freeze:

“Then when she arrested, I panicked … I haven't started the CPR yet, I said, [to another nurse] ‘press the emergency button’ that bit was automatic … and then I was like doing this [the participant mimics an action as if they were frozen/still]” – RN3.

Some participants described instances in which their emotions negatively influenced their behaviour, particularly in relation to communication with the wider MDT. In situations where colleagues were perceived as unsupportive, emotional strain occasionally resulted in a loss of professional composure:

“I was upset… I swore [at an unsupportive colleague] … you lose everything in that moment.” – RN4.

Participants reported that fear created a perceived dilemma between remaining at the bedside with a deteriorating patient and escalating care to the Rapid Response Team, which often required temporarily leaving the patient to make a telephone call. This fear sometimes led to hesitation in escalation, with some participants admitting that they delayed or refrained from escalating care despite recognising the need to act according to EWS protocols:

“So, you call help, but you don't mind, what help … as long as it's help and that its quick, I don't want to walk away from my patient when they are sick to call anyone. I would panic walking away … [describing an event when the RRT was not informed]” – RN2.

### 3.5 Theme 5: Impact on Identity, Work and Career Progression

Some participants experienced deterioration events positively, and these emotional responses appeared to enhance feelings of competence and satisfaction while strengthening their commitment to nursing and intentions to pursue a future career in the profession.

“I love to learn… I'm always learning in emergencies… it makes me confident…” – RN2.

Other participants reflected on moments when such events made them consider leaving the profession. One participant described their work withdrawal they experienced after they had escalated care to the doctors who did not attend to review a clinically deteriorating patient:

“So that time I give up… one day maybe I don't care… and the patient might be septic and be unwell.” – RN4.

Most participants stated they did not intend to leave nursing citing financial reasons and career investment. Two participants acknowledged the burden of repeatedly caring for clinically deteriorating patients and the emotional distancing strategies they employed to cope with these events made them consider leaving nursing, although external factors kept them in their posts:

“When the patient deteriorates, you think about early retirement, … It's like emotional exhaustion … I need to come back human again [after annual leave] … if I don't, I might leave [Nursing].” – RN6.

These findings are consistent with AET, which proposes that affective experiences can influence judgement‐driven behaviours, such as intentions to leave a profession or work withdrawal. While some participants described positive emotional experiences that enhanced their commitment to nursing, others reported that repeated exposure to clinically deteriorating patient events and the emotional distancing strategies used to cope with them prompted thoughts of leaving. However, participants also identified external influences, including financial commitments, and the restorative effects of annual leave, suggesting that intentions to leave were shaped not only by affective experiences but also by broader cognitive and contextual factors. This reflects the AET concept of judgement‐driven behaviours, whereby workplace attitudes and behaviours are influenced by both emotional experiences and rational evaluations.

## 4 Discussion

Optimising the recognition of and response to clinically deteriorating patients is a global patient safety priority. Despite the potential emotional consequences of this aspect of nursing work, limited research has explored nurses' affective experiences when managing patient deterioration. Informed by Affective Events Theory (AET), this study examined how workplace events associated with patient deterioration elicited emotional responses, and how individual and situational factors, such as education and clinical experience, shaped these reactions. AET also informed the development of the semi‐structured interview guide and the deductive stage of data analysis. The study further explored how these affective experiences influenced nurses' work attitudes, professional identity and career intentions.

Participants largely described negative emotional responses to clinical deterioration. This is consistent with research showing that caring for deteriorating patients often generates anxiety and moral distress (Dresser et al. 2023). Frustration commonly stemmed from limited support from members of the rapid response team (RRT), colleagues from the ward nursing team, or from unclear management plans. These findings are consistent with those of Isbell et al. (2020), who noted that negative emotions arise not only from the deterioration event itself but also from the challenges of managing it.

Our findings underscore the complex and often discordant relationship between emotion and performance (Hou and Cai 2022), with most participants reporting that feeling stressed and anxious enhanced their clinical performance, facilitating assertiveness, decisiveness and organisation. These findings are consistent with other clinical deterioration research, including a mixed methods study conducted with cardiac nurses (Woolfe Loftus et al. 2025) and research underpinned by behaviour change theory (Smith et al. 2021).

Emotional intensity appeared to trigger automatic, unconscious actions while hindering deliberate responses. One participant described “freezing” during an emergency yet still activating the cardiac arrest alarm without conscious thought. Although negative emotions typically slow cognitive processing (Hou and Cai 2022), some emotion‐driven automaticity may enable rapid action when time is critical, contrasting with slower, more reasoned decision‐making (LeBlanc et al. 2015). This dual effect aligns with dual‐process models, which propose two parallel systems influencing behaviour: a fast, automatic, non‐reflective process and a slow, effortful, reflective process (Presseau et al. 2014). Both may be needed in this situation, and emotion may shape which process nurses rely on when caring for a clinically deteriorating patient.

Participants also described how their emotional responses contributed to a reluctance to leave the patient's bedside, even when doing so delayed escalation of care to the RRT. Emotional arousal can create attentional bias, leading to a disproportionate focus on a single aspect of care and diminishing situational awareness (LeBlanc et al. 2015), for example, the need for expert support. Situational awareness refers to the ability to perceive and integrate all relevant information required for effective decision‐making and performance (Endsley et al. 2025). When situational awareness is reduced by emotional arousal, nurses may be less likely to escalate care to the RRT when doing so requires leaving the bedside, instead prioritising immediate patient care tasks without fully recognising the need for additional expert intervention.

Although participants recognised the potential value of certain emotional responses, some expressed concerns that negative emotions could adversely affect patient care. Consequently, two participants reported using coping strategies, including emotional distancing, to regulate their emotions and maintain professional performance. This reflects findings from emergency department settings, where emotional disengagement is commonly used to cope (Isbell et al. 2020). However, participants expressed concern about the long‐term consequences of emotional distancing, loss of empathy, disengagement and emotional exhaustion, core features of burnout (Rushton et al. 2015). They linked this emotional burden to thoughts of leaving the profession, echoing evidence that the tension between wanting to remain empathetic and needing to suppress emotions increases intentions to leave nursing (Kirk et al. 2021). Participants also described how the cumulative emotional burden of these experiences contributed to broader considerations of leaving the profession.

These findings align with AET which suggests that negative emotional experiences trigger coping responses. AET authors distinguished between emotion‐focused coping, which manages emotional reactions (e.g., avoidance or distancing), and problem‐focused coping, which addresses the source of stress (Weiss and Cropanzano 1996). Although emotion‐focused strategies may reduce distress temporarily, problem‐focused approaches are more likely to support long‐term adaptation.

In this study, emotional distancing appeared to provide short‐term relief from distress but did little to address the underlying challenges of managing deteriorating patients. Participants reflected that repeated reliance on this strategy could contribute to reduced empathy, emotional exhaustion, and changes in self‐perception; all features associated with burnout (Rushton et al. 2015). Therefore, supporting nurses should extend beyond individual emotion‐focused coping strategies and include problem‐focused interventions that address the sources of emotional distress, such as improving multidisciplinary team (MDT) collaboration and enhancing decision‐making support.

Participants also emphasised the importance of resilience and education in managing deteriorating patient events. Even experienced nurses reported feelings of panic when standard interventions failed, suggesting that resilience, the ability to function effectively in the face of change, may be as important as clinical experience and knowledge (Cooper et al. 2020). Although this study did not examine how resilience develops, evidence suggests that simulation‐based learning can strengthen both clinical knowledge and emotional regulation skills (LeBlanc and Posner 2022). Strategies such as mindfulness, cognitive reappraisal, and normalising emotional responses can be incorporated into simulation training while maintaining psychological safety (LeBlanc and Posner 2022). Additionally, bedside support strategies, such as structured post‐event debriefs, may reduce stress, enhance work engagement, and strengthen team support by allowing staff to process their emotions more effectively (Toews et al. 2021).

Practical solutions such as providing more portable work telephones and easier escalation of care pathways that help keep nurses at the bedside may support EWS adherence. These findings also highlight the need for nurse managers to recognise the emotional complexity involved in following EWS protocols. Moving forward, the AET could be used to inform quality improvement initiatives by offering insights into the emotional responses and behaviours of nurses (Christensen et al. 2023) and its effect on the care of deteriorating patients.

## 5 Strengths and Limitations

To our knowledge, this is one of the first studies to examine the emotional impact on ward‐based nurses of caring for clinically deteriorating patients, offering new theoretically informed insights into this high‐pressure aspect of clinical practice. One AET construct, individual disposition, did not emerge in our findings, despite evidence that personal traits can shape emotional and cognitive responses (Hou and Cai 2022). Other AET‐based studies have used quantitative methods to study traits such as optimism and extraversion (Christensen et al. 2023), suggesting that future research may benefit from mixed‐methods approaches to explore this domain further.

The researcher's insider status helped build rapport and generate rich data but also risked social desirability bias. As an active member of the hospital's RRT, participants may have overstated escalation behaviours, and the researcher's clinical experience is likely to have influenced how they analysed and interpreted the data (Bergen and Labonté 2020). Moreover, all participants had a minimum of four years' experience as registered nurses; therefore, the perspectives of newly qualified nurses were not represented. Given their comparatively limited clinical experience, newly qualified nurses may experience and respond to deterioration events differently, potentially resulting in distinct emotional reactions and coping strategies. Future studies should include larger, more diverse samples and consider mixed methods designs to deepen understanding of how emotional and dispositional factors shape care during patient deterioration.

## 6 Conclusion

Using Affective Events Theory (AET) as a guiding framework, this study examined the structure, antecedents, and consequences of ward nurses' experiences of patient deterioration events. The framework enabled exploration of how these workplace events triggered affective responses, which in turn influenced nurses' behaviours, work attitudes, career intentions and professional identity. Workplace events, including recognising a deteriorating patient, making urgent clinical decisions, assuming responsibility for factors beyond their control, and perceiving a lack of support, elicited emotions such as anxiety, fear and frustration. These affective reactions influenced perceived immediate behavioural responses, including increased assertiveness, impulsivity, temporary freezing and avoidance of escalation to non‐ward‐based teams such as the RRT.

While AET acknowledges that work attitudes and behaviours are shaped by multiple factors, including broader contextual and environmental influences such as mentorship, supervision, pay, and working conditions, emotional work events and their consequences remain important determinants of performance and retention. The findings indicate that participants perceived a relationship between the emotional experiences associated with managing deteriorating patients and both their immediate behavioural responses and longer‐term work attitudes. Therefore, understanding and addressing these affective experiences should form part of wider efforts to support nurse performance, workforce retention, and reduce burnout.

Interventions should include emotional regulation training and simulation‐based education to prepare nurses for the psychological demands of deterioration events. Quality improvement efforts, including those involving EWS, must also account for the emotional realities of bedside care to strengthen patient safety and support workforce sustainability.

## Author Contributions

Karen Davey: conceptualization, formal analysis, investigation, methodology, project administration, writing – original draft, writing – review and editing. Duncan Smith: conceptualization, methodology, writing – review and editing.

## Funding

The authors have nothing to report.

## Ethics Statement

The study received favourable opinion from the City St George's, University of London nursing proportionate research ethics committee (ID: ETH2324‐24690), the Health Research Authority (ID: 21/PR/0305), and local research and development departments from each of the three hospitals where fieldwork was conducted.

## Conflicts of Interest

The authors declare no conflicts of interest.

## Data Availability

The data that support the findings of this study are available on request from the corresponding author. The data are not publicly available due to privacy or ethical restrictions.
